# Dynamics and Sorption Kinetics of Methanol Monomers and Clusters on Nopinone Surfaces

**DOI:** 10.1021/acs.jpca.1c02309

**Published:** 2021-07-08

**Authors:** Xiangrui Kong, Josip Lovrić, Sofia M. Johansson, Nønne L. Prisle, Jan B. C. Pettersson

**Affiliations:** † Department of Chemistry and Molecular Biology, Atmospheric Science, University of Gothenburg, Gothenburg SE-41296, Sweden; ‡ Center for Atmospheric Research, University of Oulu, Oulu FI-90014, Finland

## Abstract

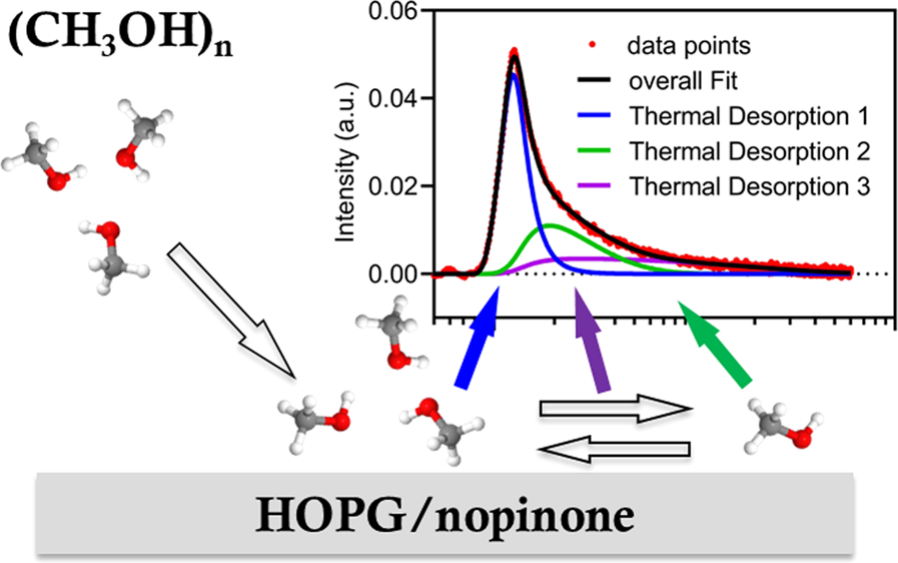

Organic–organic interactions play important roles in secondary organic aerosol formation, but the interactions are complex and poorly understood. Here, we use environmental molecular beam experiments combined with molecular dynamics simulations to investigate the interactions between methanol and nopinone, as atmospheric organic proxies. In the experiments, methanol monomers and clusters are sent to collide with three types of surfaces, i.e., graphite, thin nopinone coating on graphite, and nopinone multilayer surfaces, at temperatures between 140 and 230 K. Methanol monomers are efficiently scattered from the graphite surface, whereas the scattering is substantially suppressed from nopinone surfaces. The thermal desorption from the three surfaces is similar, suggesting that all the surfaces have weak or similar influences on methanol desorption. All trapped methanol molecules completely desorb within a short experimental time scale at temperatures of 180 K and above. At lower temperatures, the desorption rate decreases, and a long experimental time scale is used to resolve the desorption, where three desorption components are identified. The fast component is beyond the experimental detection limit. The intermediate component exhibits multistep desorption character and has an activation energy of *E*_a_ = 0.18 ± 0.03 eV, in good agreement with simulation results. The slow desorption component is related to diffusion processes due to the weak temperature dependence. The molecular dynamics results show that upon collisions the methanol clusters shatter, and the shattered fragments quickly diffuse and recombine to clusters. Desorption involves a series of processes, including detaching from clusters and desorbing as monomers. At lower temperatures, methanol forms compact cluster structures while at higher temperatures, the methanol molecules form layered structures on the nopinone surface, which are visible in the simulation. Also, the simulation is used to study the liquid–liquid interaction, where the methanol clusters completely dissolve in liquid nopinone, showing ideal organic–organic mixing.

## 1 Introduction

Organic compounds are omnipresent in the atmosphere and are continuously undergoing complex interactions and reactions.^1^ A sound understanding of atmospheric chemistry and phase transitions requires not only the individual oxidation pathways from secondary organic aerosol (SOA) precursors but also needs to take into account interactions between the multitude of different organics formed.^2^ However, the knowledge of organic–organic interactions, especially on the mechanism at a molecular level, is limited and therefore largely missing from current climate models and leads to significant uncertainties when evaluating climate changes.^3^

Biogenic volatile organic compounds (BVOCs) have been recognized as important precursors for SOA as their volatilities can be significantly reduced after oxidization.^4^ For example, β-pinene is a common BVOC emitted by coniferous vegetation and is considered as a major source of aerosol particles.^5^ As one of the major oxidation products from β-pinene,^6^ nopinone has been found in both the gas and particle phase of SOA,^7^ which motivated the recent studies on water–nopinone interactions, where nopinone was considered as a biogenic SOA proxy. Due to the cyclic structure, nopinone has a relatively high solubility in water compared to other ketones with similar sizes but linear structures.^8^ On the water surface, the uptake of nopinone was found to be reversible with a wetted-wall flow tube reactor.^9^ On the nopinone surface, the uptake of water molecules and the detailed molecular dynamics and kinetics were studied by an environmental molecular beam (EMB) technique.^10,11^ The thin coating and multilayer of nopinone show different water uptake abilities, where more water was taken up by the nopinone multilayer. Molecular dynamics(MD) simulations show that bulk diffusion is very limited and that the enhanced water uptake is instead mainly caused by water molecules that more easily find strong binding states on the nopinone multilayer surface than on the thin nopinone coating.^11^

Apart from water vapor–surface interactions, the organics–surface interactions are also important because of the abundance of organic vapors in the atmosphere and the key role of gas-to-particle partitioning of both SOA components and their precursors for SOA formation. A recent EMB-MD study shows that methanol monomers and clusters are efficiently trapped on a graphite surface, and the trapped molecules rapidly diffuse along the surface to find other clusters or form new clusters at low surface coverages.^12^ Methanol molecules form hydrogen bonds within the clusters internally, which strengthens the structure and the stability of clusters. Herrera et al.^13^ reported that small methanol clusters are preferably formed on graphite because of the interaction of hydrogen bonds. Detailed desorption kinetics of methanol from both graphite^14^ and graphene^15^ has been previously investigated. Apart from the graphite/graphene surface, various organic surfaces have also been studied by EMB,^16^ including alcohols,^17−19^ ketones,^10,11^ and carboxylic acids.^20^ Yet, interactions of organic molecules/clusters with organic surfaces have never been studied by molecular beam techniques, in spite of previous numerous studies on cluster–surface collisions^21−23^ and current adaptations of molecular/ion beam techniques to environmental and organics-related topics.^24−26^

The combination of EMB and MD has been shown to be an effective approach to study the gas uptake and sorption kinetics of gas interactions with various surfaces.^11,12,18,27^ Here, we investigate the kinetics of methanol monomers and clusters on nopinone surfaces (thin coating and multilayer) by EMB experiments and MD simulations. The interspecies interactions of individual species reveal important kinetics parameters and mixing states of the binary organic systems.

## 2 Methodology

### 2.1 EMB Experiments and Data Analysis

The EMB method is used to investigate the dynamics and kinetics of methanol interactions with nopinone surfaces. The experimental setup has been previously described in detail,^28,29^ and consists of a three-chamber differentially pumped beamline. Beam pulses are generated from a beam source, and a portion of the pulsed gas flow travels through a skimmer (diameter = 1 mm) to form a directed low-density beam of molecules in the forward direction. The nozzle of the beam source is kept at room temperature, such that no methanol condenses on the nozzle. The beam is composed of methanol and helium, where a helium (Helium HiQ 6.0, Linde plc) flow (with a He source pressure of 1 bar) passes through a methanol reservoir (assay ≥99.9%, Sigma-Aldrich, Inc.) to pick to the methanol molecules and clusters. The beam is modulated by a chopper with a frequency of 120 Hz (duty time 50%) for the short experimental time scale (10 ms) and with a frequency of 8 Hz (duty time 50%) for the long experimental time scale (60 ms). When measuring the beam using a quadrupole mass spectrometer (QMS), the most intense peak in the mass spectrum is *m*/*z* = 31 (CH_3_O^+^). Other major peaks are *m*/*z* = 33, 65, 97, 129, and 161, corresponding to H^+^(CH_3_OH)*_n_* with *n* = 1–5, i.e., the methanol clusters in the beam. It is not possible to determine the exact size of clusters due to unknown fragmentation during ionization, but the clusters are seemingly relatively small, ranging from a few molecules to tens of molecules per cluster, based on the facts of the relatively low source pressure, the shape of the nozzle that is not optimized for cluster production, and the relatively similar intensities of monomers and clusters.

The measured time-of-flight (ToF) distributions of *m*/*z* = 31 (CH_3_O^+^) and *m*/*z* = 33 (H^+^CH_3_OH) intensities show that the clusters travel with lower velocities than monomers, as known from earlier studies.^12^ The beam is supersonic, and the estimated mean velocity for monomers is about 1510 m/s, corresponding to a kinetic energy (KE) of 0.38 eV. The beam passes through a grated opening and impacts the experimental surfaces with an incident angle of 45° with respect to the surface normal. The beam pulses then collide with a surface centered in an environmental chamber (highest experiment pressure ≈ 10^–2^ mbar), and the outgoing flux is measured by a rotatable differentially pumped QMS for ToF measurements. Ions generated by electron bombardment in the QMS are detected by a multichannel scaler with a dwell time of 10 μs. The studies presented here are performed with a highly oriented polycrystalline graphite (HOPG) substrate surface (12 × 12 mm surface, Advanced Ceramics Corp., grade ZYB), which is cleaned by keeping it at 600 K before and after experiments. The nopinone surfaces are prepared by dosing nopinone ((1*R*)-(+)-nopinone, 98% Sigma-Aldrich Co.) vapor through a leak valve, where the nopinone multilayer is maintained at a thickness of ∼1 μm. The properties and thickness of the condensed nopinone layer are monitored by the helium in the beam and a laser (670 nm) interferometry. The thickness of the nopinone multilayer is monitored by the laser, whereas the thin nopinone coating on HOPG cannot be seen by the laser but is detected by helium scattering.^11^ Both nopinone thin coatings and nopinone multilayers are studied, where the thin coating is analogous to the coating layer on the soot surface and the multiple layer can be considered as the model system for a nopinone SOA particle.

The ToF distributions are fitted to resolve the kinetics and dynamics of the interactions between the impacting methanol molecules/clusters with the nopinone surfaces. Typically, impinging molecules can be scattered inelastically or thermally trapped, thus these components are sought for in our analysis. Nonlinear least squares fits are carried out to deconvolute the inelastic scattering (IS) and thermal desorption (TD) components. The IS component is represented by a velocity-dependent function,^11,19^
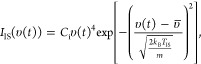
1where *C*_i_ is a scaling parameter, υ is the velocity calculated from the molecular arrival time, υ̅ is the average velocity, *k*_B_ is the Boltzmann constant, *m* is the molecular mass of methanol, and *T*_IS_ is a free parameter representing the IS velocity spread. Note that the electron-impact ionization process in the employed mass spectrometer is velocity-sensitive and an additional transmission factor (a function of υ) is taken into account in the classical velocity-dependent function.^30,31^

The TD distributions are each a combination of two components: (i) a velocity distribution that relates desorption to molecular excitation based on the surface temperature,
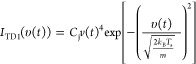
2and (ii) another distribution related to the desorption rates,
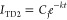
3where *C*_j_ is a free scaling factor, *T*_s_ in eq 2 is the surface temperature, *k* is the fitted desorption rate coefficient, and *t* is time. *I*_TD1_ shows the velocity spread of the TD flux, and *I*_TD2_ accounts for the exponential decay of ToF distributions. Thus, the TD distributions are calculated as a convolution of these two components.

### 2.2 MD Simulations

MD simulations are performed to characterize methanol monomer and cluster collisions with a solid nopinone surface. The classical mechanics GROMOS force field^32^ optimized for small molecules in condensed phases is employed to model a nopinone crystal. The force field is implemented in the GROMACS package^33^ with the help of the Automated force field Topology Builder (ATB) database.^34^ The original ATB GROMOS charges are unable to reproduce melting processes; therefore, a new set of charges is calculated based on ab initio calculations. RESP^35^ point charges fixed on the atomic position are fitted to best reproduce the electrostatic field induced by the electronic density of the isolated nopinone molecule calculated on BLYP-D3 level of theory while having electrons spanned over the DZVP basis set.

The equations of motion are integrated using the leap-frog approach^36^ and LINCS constrain algorithm,^37^ which makes it possible to use a 2 fs time step. A cutoff distance of 1.8 nm is applied for the short-range interactions, and long-range electrostatic interactions are treated using the particle mesh Ewald method.^38^ Nopinone crystal temperature is held at the desired value using the V-rescale algorithm^39^ with a coupling time of 0.1 ps.

The nopinone crystal is modeled based on the powder X-ray diffraction data by Palin et al.^40^ The crystal structure is freely available at the Cambridge structural database.^41^ An infinite crystal is created by duplicating a unit cell in *x*-, *y*-, and *z*-directions. After a minimization with the steepest descent algorithm, the crystal is equilibrated in the NPT ensemble at 220 K for 10 ns. The simulation system consists of ∼10,000 atoms placed in an orthorhombic box stretched by *a*, *b*, and *c* vectors sized 3.91 × 4.18 × 7.53 nm. The relaxed crystal is then used to form a slab considering the most energetically favorable exposed crystal surface; thus, the crystal is cut between two nopinone bilayers. The simulation box is then extended up to 2 nm along the *z*-direction on each side to avoid image interactions between the slabs. After producing the nopinone solid slab, methanol interactions with the nopinone surface are studied at 220 K. Simulations are designed to resemble the experimental conditions. Monomers and methanol clusters are present in the beam, therefore both cases are modeled. Methanol is also modeled with the GROMOS force field^32^ and refined RESP^35^ point charges are applied as previously explained for nopinone.

First, to model monomer collisions, single methanol molecules are sent toward the surface with an incident KE equal to 0.48 eV (1700 m s^–1^) and with an incident angle of 45° with respect to the surface normal direction. Methanol is decoupled from the thermostat to avoid influencing collision dynamics. The initial (*x*, *y*) positions of methanol molecules are randomly chosen at a distance of 1 nm from the surface and 2000 trajectories are propagated at each studied surface temperature. Methanol clusters on the nopinone surface system are also investigated. Initially, 10 methanol molecules are packed in a cluster and equilibrated at 180 K in the NVT ensemble. Thereafter, the cluster is placed 1 nm above the solid nopinone surface at 220 K and consecutively sent toward the surface at a velocity of 1700 m s^–1^ and an incident angle of 45° with respect to the surface normal direction while being decoupled from the thermostat.

## 3 Results and Discussions

### 3.1 EMB Experiments

#### 3.1.1 Monomer and Cluster Beam Profiles

**Figure 1:**
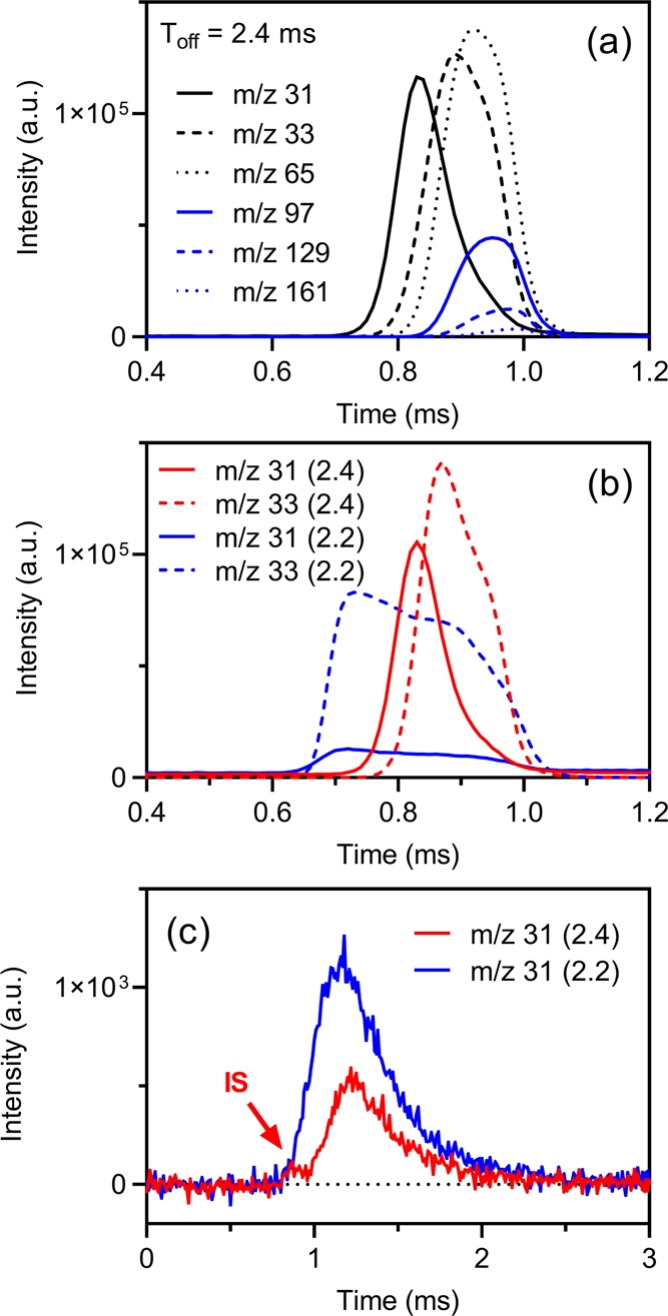
(a) Beam profiles of monomers and clusters of different sizes; (b) beam profiles of *m*/*z* = 31 and *m*/*z* = 33 for the monomer beam (*T*_off_ = 2.4 ms) and the cluster beam (*T*_off_ = 2.2 ms); and (c) ToF of the monomer flux from graphite at 200 K, using the monomer beam (*T*_off_ = 2.4 ms) and the cluster beam (*T*_off_ = 2.2 ms). The incident and detection angles are both 45°.

The molecular beam used in EMB experiments contains methanol monomers and methanol clusters of various sizes.^12^ The beam composition is varied by changing the beam source off time (*t*_off_ = 2.2 or 2.4 ms), which shifts the beam with respect to the chopper opening. Figure 1a shows the profiles of the beam recorded by a ToF mass spectrometer when *t*_off_ = 2.4 ms, where both clusters and monomers appear as peaks. This feature allows for the study of the interactions between monomers and the surfaces. Note that the monomers and clusters are represented by *m*/*z* = 31 and 33, respectively, but the criteria to distinguish them are the velocity differences shown in Figure 1a. All the peaks have independent shapes, indicating that the clusters only contributed to their own primary *m*/*z* but not to others, otherwise the lower *m*/*z* values would show contributions from the higher *m*/*z* values. The molecular velocities depend on the monomer or cluster masses, so the lightest mass, i.e., monomers (*m*/*z* = 31), appears at the beginning of the ToF spectrum and the heavier clusters arrive later. Another beam setting is the cluster beam (*t*_off_ = 2.2 ms), and the beam profile is shown in Figure 1b. In this case, the beam is dominated by clusters with a square-like beam shape, which enables accurate fittings to the ToF results. Hence, in the desorption kinetics section, this cluster beam is used.

Figure 1c shows an example of the methanol flux from a 200 K graphite surface after beam–surface collisions, where the monomer and cluster beams are compared. For the cluster beam (*t*_off_ = 2.2 ms), the flux appears as a single peak, while for the monomer beam (*t*_off_ = 2.4 ms), there is an additional component appearing at the beginning of the ToF spectrum. This is because of the presence of a fraction of monomers that travel faster than clusters (Figure 1a), and these monomers result in an IS component. The comparison of these two fluxes thus shows that methanol monomers are scattered from the surface while the clusters are not. Under the experimental incident KE, no cluster flux (*m*/*z* 33 and 65) is detected from any surfaces, indicating that methanol is scattered or desorbs only as a monomer.

#### 3.1.2 Inelastic Scattering and Thermal Desorption

**Figure 2:**
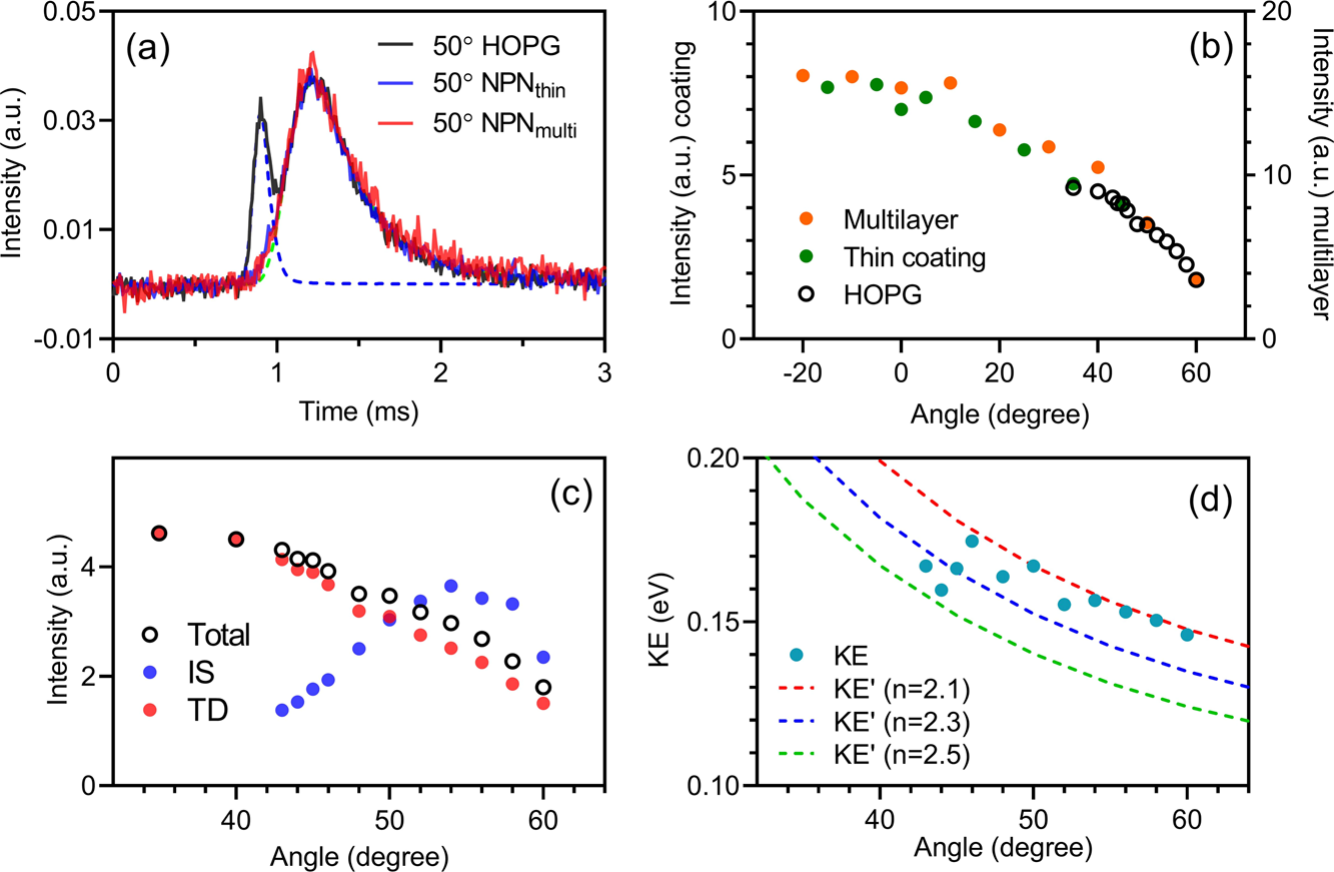
(a) ToF spectra of the methanol flux from graphite, nopinone coating, and nopinone multilayer surfaces after monomer beam collisions. The surface temperatures are 210 K, and the detection angle is 50° with respect to the surface normal. (b) Angular distributions of the total methanol flux from graphite, nopinone coating, and the nopinone multilayer at 210 K. (c) Angular distributions of IS and TD components, and the IS intensity is multiplied by a factor of 8 to be showed in the same scale. (d) Angular distributions of measured KE of IS molecules and three reference curves (KE’). The reference curves have constant parallel KE dispersion (by a factor of *n*). The perpendicular KE is also scaled by *n*, but it is variable and responsible for the angular dependence.

When methanol monomers impinge on the surfaces of nopinone coating or the nopinone multilayer, the scattered component is significantly different compared to that from a graphite surface. Figure 2a shows that the IS component (shown in blue dashed lines) from graphite vanishes from its original position in the nopinone cases. This indicates that methanol molecules transfer energy efficiently with the nopinone surfaces, which is comparable to the case of water molecules impinging on nopinone surfaces that has been studied previously.^10,11^ The methanol–nopinone surface interactions have also been investigated by MD simulations, where both IS and fast TD are found (Figure S2). Other than that, the desorption components remained almost identical in the three cases. Because the IS components are subtle, the angular distribution of total flux from nopinone surfaces are essentially cosine-shaped (Figure 2b), which is a characteristic for TD components.

**Figure 3:**
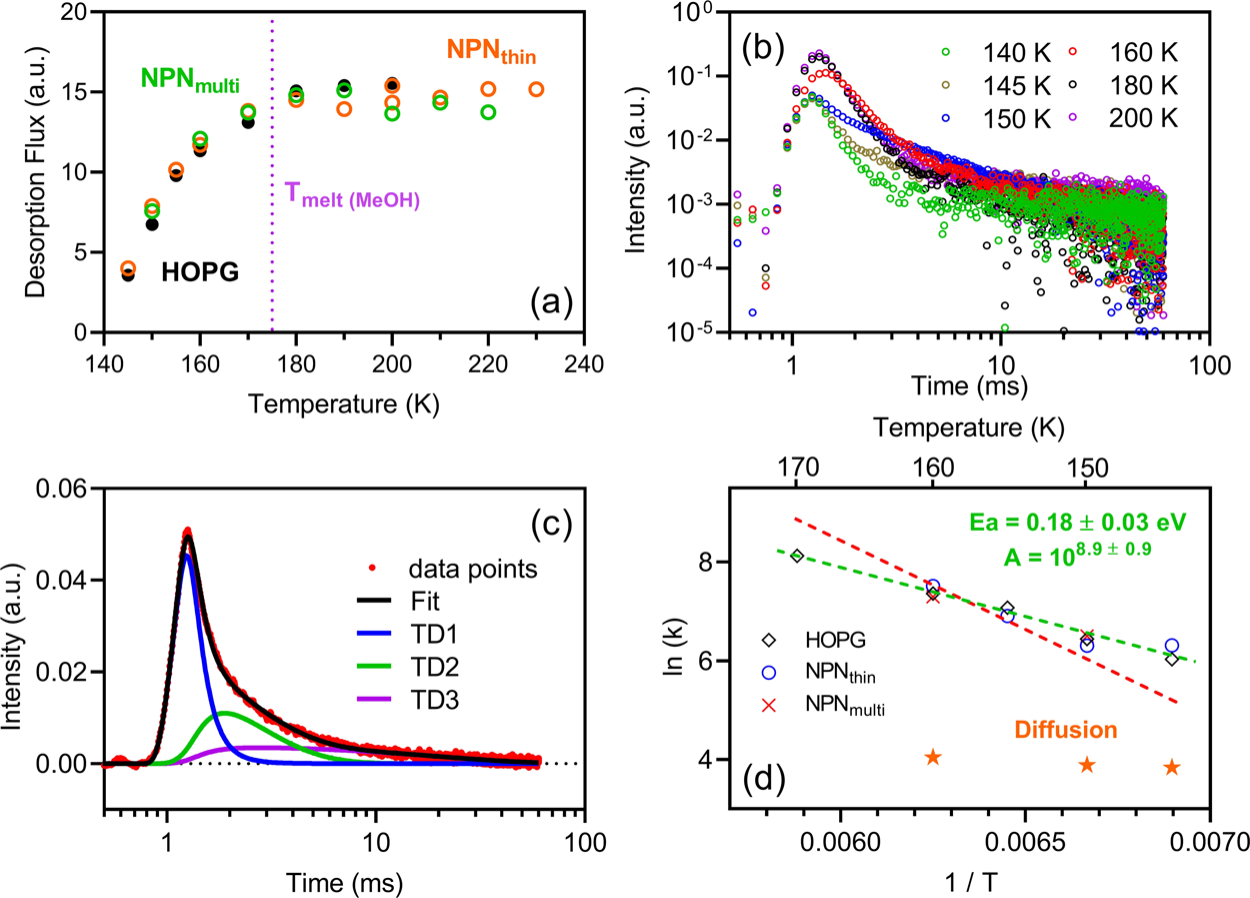
(a) Temperature-dependent desorption flux from graphite, thin nopinone coating, and the nopinone multilayer. The intensities have been normalized to beam intensities. The melting point of bulk methanol is marked as the purple dotted line. The experimental time scale is 10 ms. Both the incident angle and detection angle are 45°. (b) ToF of long experiments (60 ms) at various temperatures. (c) ToF measured at 150 K, fitted by three TD components. (d) Arrhenius plot for the TD2 component at the temperature range between 140 and 170 K. The green dashed line is the best fit to the data points, where *E*_a_ = 0.18 ± 0.03 eV and *A* = 1·10^8.9 ± 0.9^. The red dashed line is the best fit with the assumption of *A* = 1·10^13^.

Figure 2c shows the angular distributions of IS and TD flux intensity from the graphite surface. The IS intensity peaked at around 54° (*c.f.*, incident angle = 45°), and the IS peaked at wide angles, which is common for molecules scattering on smooth surfaces.^42^Figure 2d displays the variation of KE (0.14–0.18 eV) of the IS molecules, accounting for 37–47% of the initial KE. The KE decreases as the scattering angle increases, which is because the graphite surface is smooth on the molecular level, such that the parallel momentum of impinging molecules is largely conserved. Therefore, the molecules of higher KE are those conserving a higher perpendicular momentum. Then, the higher perpendicular momentum and steady parallel momentum gives higher KE and a lower angle with respect to the normal (see the reference curves in Figure 3d).^42−44^ Note that the trend of high KE at smaller angles with respect to the normal may be altered by other parameters, such as the structure of incident molecules, incident angle, and initial KE.^45,46^ The IS components cannot be fitted for the observation angles <43° because the signal is too small.

#### 3.1.3 Desorption Kinetics

The desorption kinetics is studied by using the cluster beam for its more square-shaped beam shape. The desorption flux from the graphite, thin nopinone coating, and nopinone multilayer surfaces shows similar temperature dependence. Figure 3a shows that the desorption fluxes increase with temperature below 180 K. Above 180 K, the desorption intensities do not change with temperature, indicating that all trapped methanol molecules have desorbed within the detection time scale (10 ms). It is interesting to see that neither the nopinone coating nor the multilayer influences the methanol uptake compared to the graphite surface. The insensitivity of all three investigated surfaces suggests that the desorption processes are dominated by internal methanol–methanol interactions within clusters. Notably, the melting point of methanol is ∼175 K (marked with a dashed purple line in Figure 3a), which separates the desorption flux into a temperature-sensitive region and a temperature-insensitive region.

The detection time scales may potentially influence the quantification of the TD fractions, especially at lower temperatures.^47^ To account for this, longer experiments with 60 ms scanning time were carried out and the ToF spectra are shown in Figure 3b. Apparently, at most temperatures the desorption does not end by 60 ms, which confirms the existence of slow desorption channels. Three TD fits are used to fit the 60 ms ToF spectrum at the temperature range between 140 and 170 K (Figure 3c). Two TD fits are also attempted but there were always noticeable residues between fittings and actual data points, showing that it is necessary to have three TD components (see Figure S1 for example). Note that the ToF at temperatures higher than the methanol bulk melting point, i.e., 180 and 200 K, cannot be fitted using three TD components but by only one (the fastest desorption, TD1). The absence of the other two TD components is likely due to the solid–liquid phase transition of methanol on the surface, i.e., the TD2 and TD3 are processes occurring on nonliquid surfaces.

As the rates of TD1 are always beyond the EMB detection limit (EMB time resolution = 10 μs, i.e., resolvable *k* must be ≤10^5^ s^–1^), only the resolvable rates of TD2 and TD3 are presented in the Arrhenius plot (Figure 3d). The slow TD component (TD3) is likely associated with diffusion-related processes due to the weak temperature dependence. As for the intermediate desorption component (TD2), no differences are found among the three kinds of surfaces. The activation energies are around 0.18 ± 0.03 eV with a preexponential factor (*A*) of 1·10^8.9 ± 0.9^. Such low activation energy is comparable to the binding energy of the methanol monomer on graphite,^48^ but the low *A* value indicates that the yielded activation energy is a result from complicated processes rather than a first-order desorption. For comparison, by artificially assigning the preexponential factor to the typical value for first-order desorption (*A* = 1·10^13^), the activation energy obtained from the constrained best fitting is then 0.31 eV (red dashed line), but it is clearly deviating from the experimental data points.

### 3.2 MD Simulations

#### 3.2.1 Collision Dynamics

**Figure 4:**
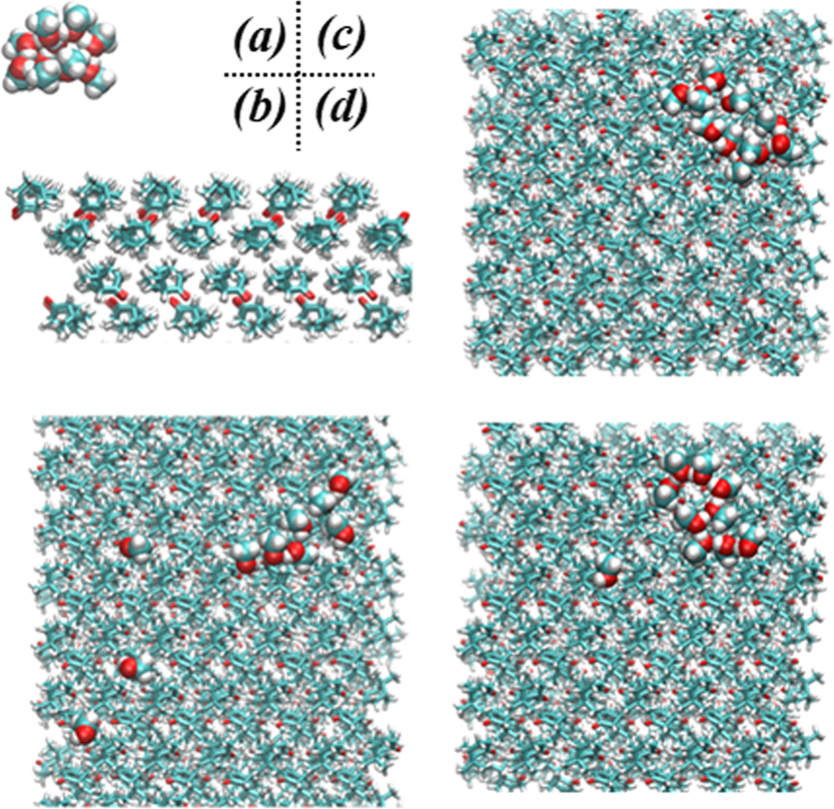
Snapshots of a 10-molecule methanol cluster colliding with the solid nopinone surface at 200 K. Snapshot taken at (a) *t* = 0 ps; (b) *t* = 24 ps; (c) *t* = 108 ps; and (d) *t* = 154 ps.

The methanol–nopinone system is modeled by classical MD, where both monomer–surface and cluster–surface interactions are simulated. The nopinone crystal is constructed based on the experimental data,^40^ which is characterized by a bilayer structure having functional groups directed inside the bilayer (Figure 4a). The molecules within bilayers cohere through relatively week hydrogen bonds between carbonyl groups from one side and hydrogens atoms from the surrounding molecules.^40^ Weak van der Waals forces between bilayers are responsible for overall crystal structure cohesion. The melting point of nopinone has been experimentally determined to 260 K.^40^ In this work, the investigated temperature is well below the melting point of bulk nopinone, i.e., the simulations are conducted at 220 K, so nopinone has a crystalline structure. The nopinone surface is characterized by well-ordered hydrocarbon groups where carbonyl groups were generally inaccessible. The appearance of hydroxyl groups on the surface may be considered as surface defects, which appears as one of the surface molecules rotate for 180° around one of the surface axes. Such hydrophilic sites on the surface influence the desorption dynamics of incoming molecules. This is especially evident for small molecules, like water,^11^ which can rapidly diffuse on a smooth nopinone surface and eventually bind strongly for the hydroxyl site.

Figure 4 presents snapshots from simulations taken on critical moments during methanol cluster–surface collisions, which mimics the moments when the beam methanol cluster collides the nopinone surface. Figure 4a shows the initial conditions of the simulations, where a 10-molecule cluster is placed 1 nm above the surface. Just after colliding with the surface and during the first 20–30 ps, the cluster shatters and fragments on the surface (Figure 4b). Evidently, there is a significant number of methanol monomers loosely bound to the nopinone surface (Figure 4c), which can easily undergo TD. In the next step, methanol molecules that do not undergo desorption within a period of ∼100 ps from impacting the surface form clusters on the surface again. Methanol molecules diffuse on the surface efficiently (diffusion coefficient ≈ 5.35 × 10^–5^ cm^2^/s), which allows them to quickly find other methanol molecules or clusters and bind strongly to these. This is comparable to methanol monomer diffusion on the graphite surface, even at very low methanol-surface coverages.^12^ Molecules inside clusters are more strongly bound and therefore not able to desorb directly. The molecules on the cluster edges are relatively weakly bound, and they can either directly desorb from clusters or go through a two-step process where they first detach from clusters (Figure 4d) and then desorb as monomers.

#### 3.2.2 Cluster Evolution

**Figure 5:**
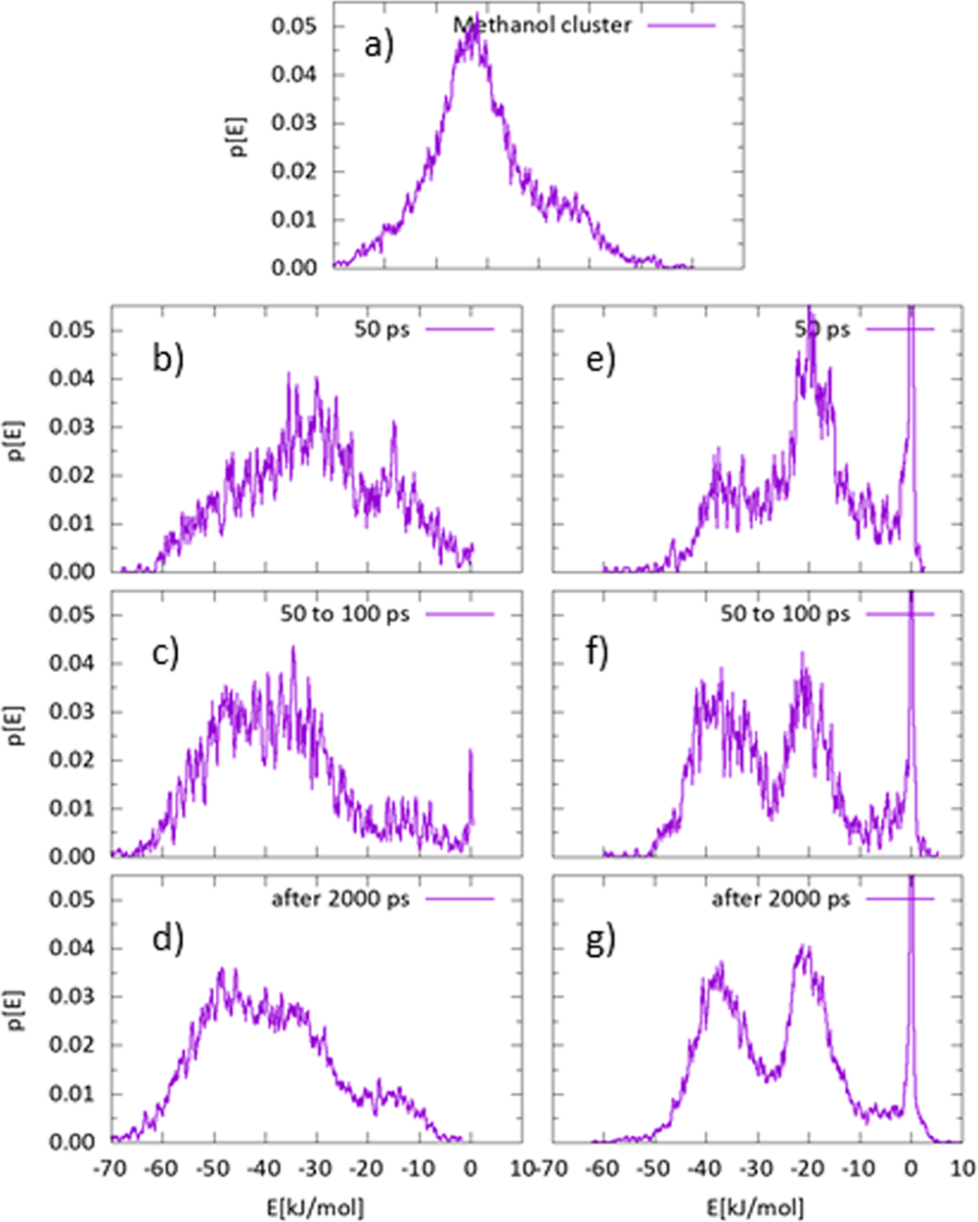
Probability distributions retrieved by histograming instantaneous binding energies between individual methanol molecules with surrounding molecules. Panel (a) shows distribution inside isolated methanol clusters. On the left panel, from (b) to (d), are distributions corresponding to binding energies between methanol molecules and the surrounding system including the nopinone surface. On the right side, from panels (e) to (g), the nopinone surface is omitted from calculation of binding energy, thus showing exclusively interactions between methanol molecules. The time window for which distribution is calculated is visible as a legend in the upper right corner of each individual plot.

The evolution of methanol clusters over time is presented from the aspect of binding energy distribution shown in Figure 5. The energy is calculated as the instantaneous interaction energy of one methanol molecule with the whole surrounding system including the nopinone surface and other methanol molecules (left panel) and without the nopinone surface (right panel). The distribution in the top panel is corresponding to isolated methanol clusters, which serves as a reference for the other two cases. All distributions shown in Figure 5 are characterized by a broad energy range indicating the complex nature of the system, which results in multistep dynamics of clustering. This agrees with the experimentally determined kinetics parameter (Figure 3), which indicates complex desorption mechanisms of methanol from nopinone surfaces. Figure 5b shows the snapshot ∼50 ps after the collisions and the weakly bounded states are visible, as the energy distribution is shifted to the lower energies. The cluster undergoes fast rearrangements as seen in Figure 5c as the distribution is shifted to higher binding energies again. At approximately 2 ns after surface impact, methanol clusters are stable on the surface. The distribution profile in Figure 5d shows the multiple components corresponding to several binding states in the clusters.

For comparison, the right-hand side panels show the energy distributions when the nopinone surface is omitted; thus, only the methanol–methanol interactions are visible. Apparently, when there is no surface present, the energy distribution is more sharply peaked at −40 kJ mol^–1^ (∼2 hydrogen bonds) and −20 kJ mol^–1^ (∼1 hydrogen bond), respectively (Figure 5g), revealing two typical conformation configurations. One of these is stronger, inside of the cluster and the other, which is seen on the edges, is more probable to desorb. The difference between the left and right panels indicates the influence of the nopinone surface on the cluster-binding states. The presence of a surface strengthens the binding energy of the methanol molecules due to the methanol–nopinone interactions, and the smoothing effect of the surface indicates the complexity of the methanol cluster on the nopinone surface system. Nevertheless, inside clusters, the methanol–methanol interaction energy is dominant in the overall energy distribution, especially when the cluster is equilibrated on the surface, and this supports the conclusion statement that desorption dynamics are mainly driven by internal cluster methanol–methanol interactions.

#### 3.2.3 Temperature Effects

**Figure 6:**
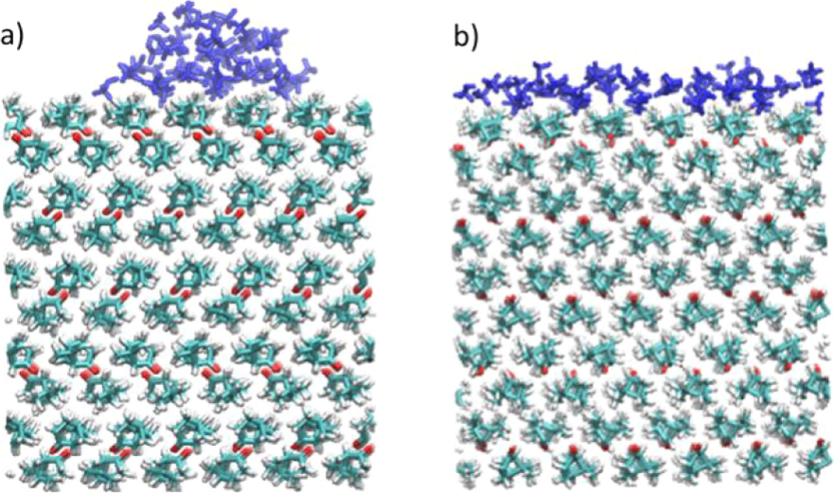
Configurations of a methanol cluster (in blue) on top of solid nopinone surfaces (green, red, and white atom representation) at temperatures (a) *T* = 100 K and (b) *T* = 150 K.

Methanol desorption from each of the investigated surfaces (a graphite surface, a thin nopinone coating on graphite, and a solid nopinone surface) shows a uniform temperature dependence (Figure 3a), indicating that all the surfaces either have similar or negligible influences on the desorption processes. To understand the temperature dependence of methanol desorption from the nopinone surface, a methanol cluster consisting of 60 molecules is placed above the nopinone surface and equilibrated at different temperatures. Two distinct configurations of methanol clusters on top of the nopinone surface at two different temperatures (100 and 150 K, respectively) are shown in Figure 6. At the lower temperature (Figure 6a), the methanol cluster retains a compact shape, and the methanol–methanol internal interactions (binding energy = 30.8 kJ mol^–1^) are stronger than the methanol–surface interactions (binding energy = 8 kJ mol^–1^). At the higher temperature (Figure 6b), the methanol molecules spread on the nopinone surface and the methanol–surface interactions become more significant (binding energy = 25 kJ mol^–1^) while the internal methanol–methanol interactions are weakened (binding energy = 13 kJ mol^–1^). The total binding energies of methanol molecules are comparable in the two cases, i.e., 40 kJ mol^–1^ at the lower temperature and 38 kJ mol^–1^ at the higher temperature. The observed enhancement of desorption in the EMB experiments at higher temperatures may be attributed to the increased wettability by methanol on nopinone. This accelerates the monomer detachments from clusters, which may lead to an increase in desorption. Temperature increase eventually leads to cluster destruction, whereby the stronger interactions from ∼2 hydrogen bond conformations vanish and in turn make methanol monomers free to desorb from the nopinone surface. Thus, the surface acts as a boundary which interacts relatively weakly with methanol molecules and restricts the movements of trapped molecules.

#### 3.2.4 Liquid–Liquid Mixing

**Figure 7:**
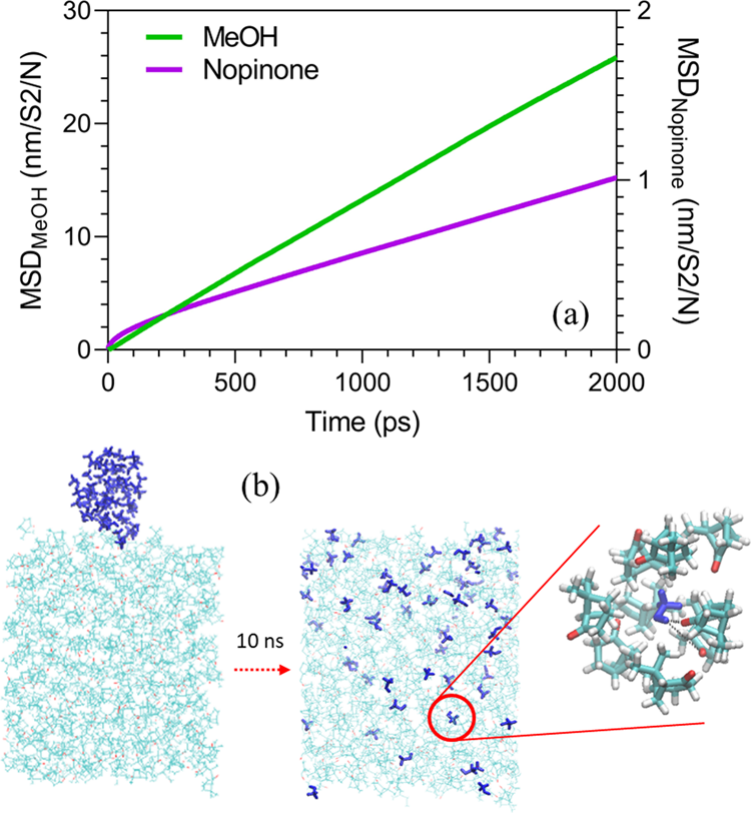
(a) MSD evolution for nopinone and methanol during melting. (b) Snapshots of nopinone and methanol before and after melting in 10 ns simulation time.

So far, only solid nopinone has been studied, the organic–organic system does not display any signs of mixing between methanol and nopinone, even though methanol species could well wet the solid nopinone surface. The mixing of liquid methanol and nopinone is, however, interesting to investigate further, particularly for its implications for SOA.^49−51^ Due to technical and physical limitations of the current EMB setup, it is not possible to experimentally investigate liquid–liquid interaction. Instead, the case of methanol clusters (*n* = 60) adsorbing on a liquid nopinone surface is simulated in MD. Liquid nopinone is obtained by applying simulated annealing all the way to 330 K, and the nopinone bulk is then equilibrated at 270 K. Figure 7a shows that the mean square displacements (MSD) of both methanol and nopinone are constantly growing over time at 270 K. The increasing MSD of methanol and nopinone indicates the fine mixing between the two compounds, with a methanol diffusion coefficient *D*_methanol_ = (1.64 ± 0.14) × 10^–5^ cm^2^ s^–1^. Figure 7b shows the initial and final snapshots, with an enhanced insert showing a methanol molecule and its neighboring nopinone molecules. Methanol clusters are dissolved in nopinone efficiently and methanol monomers are able to quickly find strong binding sites and form hydrogen bonds with nopinone molecules (Figure 7b). The mixing ability of methanol and nopinone influences several key physiochemical properties of their mixtures, such as the viscosity which in turn affects the further gas-to-particle partitioning of components to the mixture, as well as cloud droplet and ice nucleation properties and heterogeneous chemistry of the system.^52−55^

## 4 Conclusions

The methanol–nopinone interactions on surfaces are investigated by EMB experiments and MD simulations. In the EMB experiments, methanol monomers and clusters are sent to collide with graphite, nopinone coating, and nopinone multilayer surfaces in the temperature range between 145 and 230 K. Methanol monomers are efficiently scattered from the graphite surface, and the scattering is significantly suppressed from nopinone surfaces, indicating a more significant energy transfer between methanol and nopinone surfaces. For methanol cluster–nopinone surface interactions, no significant differences in collision dynamics and desorption processes are observed, suggesting that the desorption is governed by methanol–methanol interactions (via hydrogen bonds). Most trapped methanol desorb within 10 ms at temperatures of 180 K and above. At temperatures <180 K, the desorption rate decreases and a long experimental time scale (60 ms) is used to resolve the desorption. Three desorption components are identified. First, the fast component is beyond the detection limit and therefore cannot be resolved. The intermediate TD component exhibits a multistep desorption characteristic and has an activation energy of *E*_a_ = 0.18 ± 0.03 eV, with a preexponential factor *A* = 10^8.9 ± 0.9^. The slow desorption shows a weak temperature dependence, indicating that it may be associated with diffusion processes as methanol molecules exchange between clusters. The MD results show that upon collision the methanol clusters shatter, and the shattered fragments quickly diffuse and recombine to clusters. The desorption involves a series of processes, including detaching from clusters and desorbing from the nopinone surfaces as monomers. The simulations reveal that at lower temperatures methanol form compact cluster structures while at higher temperatures the methanol molecules form layered structures on the nopinone surface. In addition, MD simulations are used to study the liquid–liquid interactions between methanol and nopinone, where the methanol clusters completely dissolve in liquid nopinone, showing ideal organic–organic mixing.

## Data Availability

The data that support the findings of this study are available from the corresponding author upon reasonable request.

## Supporting Information Available

Comparison of fittings with two and three TD components and MD angular distribution of methanol monomers from the nopinone surface at 220 K (PDF)
