# Supplementary material for: Dynamics and Sorption Kinetics of Methanol Monomers and Clusters on Nopinone Surfaces
Source: J Phys Chem A. 2021 Jul 8;125(28):6263–72. doi: 10.1021/acs.jpca.1c02309 (PMC8311642; doi:10.1021/acs.jpca.1c02309)
Supplement: Supplementary file 1 — jp1c02309_si_001.pdf [file jp1c02309_si_001.pdf]

Supporting Information

for

Dynamics and Sorption Kinetics of Methanol Monomers and Clusters on Nopinone Surfaces

Xiangrui Kong[1*], Josip Lovrić[1], Sofia M. Johansson[1], Nønne L. Prisle[2], and Jan B. C. Pettersson[1*]

[1]*Department of Chemistry and Molecular Biology, Atmospheric Science, University of Gothenburg, SE-41296, Gothenburg, Sweden*

[2]*Nano and Molecular Systems research unit, University of Oulu, 90014, Oulu, Finland*

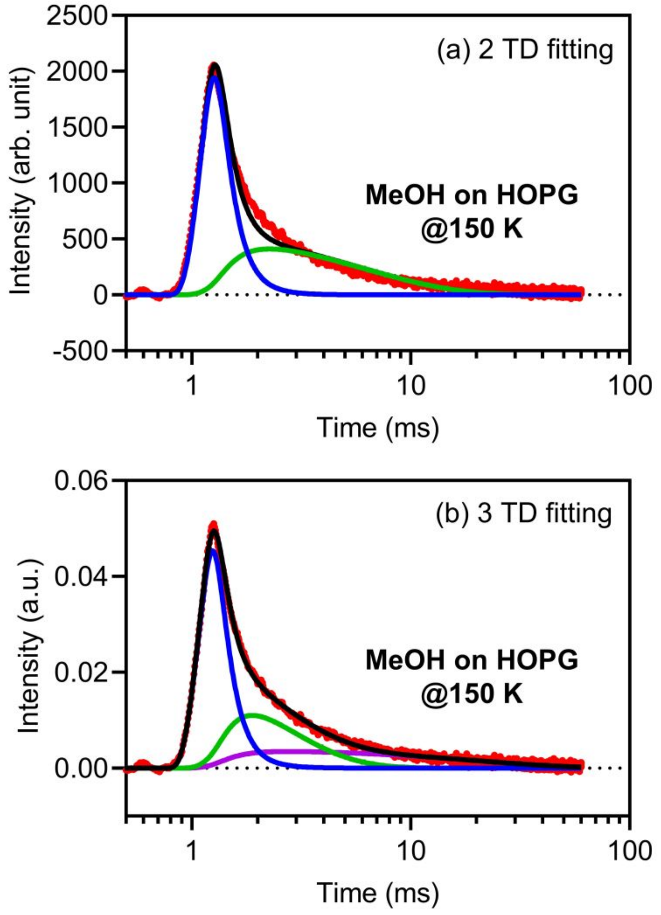

Figure S1 Comparison of 2 TD fitting and 3 TD fitting on a same ToF, where there was always significant residue between fitting and actual data points when the 2 TD fitting is tried.

In the MD simulation, methanol monomers were randomly sent toward the solid nopinone surface. Within time window of 120 ps, 40% of the colliding monomers end up with desorption. Smaller fraction of these molecules scatters inelastic and most of the molecules are thermally desorbed. This is clearly visible in Figure S2, which depicts the angle resolved methanol monomer flux from the nopinone surface. The total outgoing flux is represented by purple bins, and the green line represent thermal desorption component fitted to cosine distribution. The IS component, visible as a blue line, is reproduced subtracting TD component from the total flux. The depicted IS distribution has a maximum on a wider angle (~55°) than direction of specular direction, which is typical for IS from smooth surfaces.

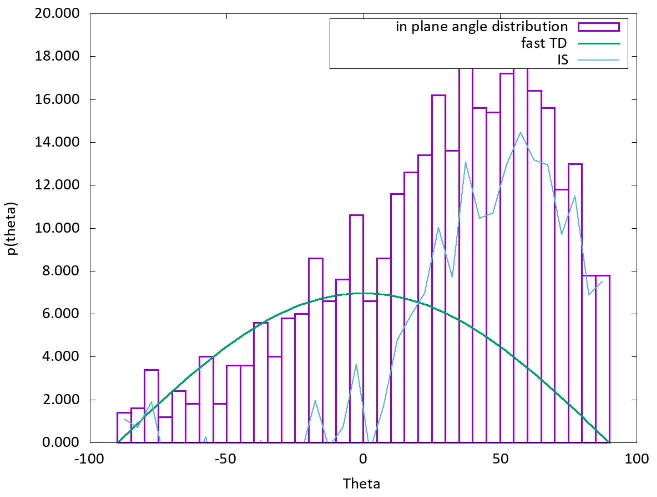

Figure S2 MD angular distribution of outgoing methanol monomers from solid nopinone surface at 220 K. Purple bins correspond to total flux, green line represents fast TD component reproduced as a fit on to total flux negative angle and a blue line is difference between total flux and fast TD i.e., showing IS component.
